# Role of Heme Oxygenase in Gastrointestinal Epithelial Cells

**DOI:** 10.3390/antiox11071323

**Published:** 2022-07-05

**Authors:** Reiko Akagi

**Affiliations:** Department of Pharmacy, Faculty of Pharmacy, Yasuda Women’s University, 6-13-1 Yasuhigashi, Asaminami-ku, Hiroshima 731-0153, Japan; akagi@yasuda-u.ac.jp; Tel.: +81-080-4600-4394; Fax: +81-82-878-9540

**Keywords:** heme oxygenase-1, heat shock protein 70, carbon monoxide, bilirubin, gastrointestinal epithelial cell, barrier function, oxidative stress

## Abstract

The gastrointestinal tract is a unique organ containing both vascular and luminal routes lined by epithelial cells forming the mucosa, which play an important role in the entry of nutrients and act as a selective barrier, excluding potentially harmful agents. Mucosal surfaces establish a selective barrier between hostile external environments and the internal milieu. Heme is a major nutritional source of iron and is a pro-oxidant that causes oxidative stress. Heme oxygenases (HOs) catalyze the rate-limiting step in heme degradation, resulting in the formation of iron, carbon monoxide, and biliverdin, which are subsequently converted to bilirubin by biliverdin reductase. In gastrointestinal pathogenesis, HO-1, an inducible isoform of HO, is markedly induced in epithelial cells and plays an important role in protecting mucosal cells. Recent studies have focused on the biological effects of the products of this enzymatic reaction, which have antioxidant, anti-inflammatory, and cytoprotective functions. In this review, the essential roles of HO in the gastrointestinal tract are summarized, focusing on nutrient absorption, protection against cellular stresses, and the maintenance and regulation of tight junction proteins, emphasizing the potential therapeutic implications. The biochemical basis of the potential therapeutic implications of glutamine for HO-1 induction in gastrointestinal injury is also discussed.

## 1. Introduction

The gastrointestinal tract is the largest interface between the body and the environment. It plays a critical role in the absorption of nutrients as well as in the development of multiple organ damage, both as a contributor to systemic inflammation and as a site of end-organ injury [1,2]. The surface of the intestinal tract comprises a single monolayer of intestinal epithelial cells. They express special transporters that absorb nutrients and form a barrier restricting free exchange across the paracellular space.

Heme is an essential nutritional source of iron and is mostly absorbed in the proximal half of the duodenum; its absorptive capacity decreases in the distal part of the small intestine [3]. The heme absorbed into enterocytes is degraded by heme oxygenases (HOs) [4], and HO-1 is a major 32 kDa heat shock protein (HSP32) [5]. We recently reported that the high expression of HO-1 in trophoblastic cells of the human placenta, which is also an absorptive tissue, plays a vital role in the iron supply system during the second trimester, supporting fetal development [6].

It has been widely accepted that HO-1 plays a significant role in protecting against inflammatory and oxidative tissue injuries [7], and is a therapeutic target in oxidative tissue injury. In various models of oxidative tissue injury, HO-1 induction confers protection to tissues against further damage by removing the pro-oxidant heme or by the antioxidative, anti-inflammatory, and/or anti-apoptotic actions of one or more of the three products of its catalytic activity, including carbon monoxide (CO), biliverdin/bilirubin, and iron [8]. HO-1 is a Phase II detoxifying enzyme that is constitutively expressed in the normal gastric and colonic mucosa obtained from patients; however, when inflamed, it is differentially expressed in these tissues [9], suggesting that gastrointestinal HO-1 expression is regulated in a condition-specific manner. We previously reported that high levels of intracellular free heme result in intestinal barrier disruption, which is reversed by HO-1 induction [10].

In this review, the vital role of HOs in the iron supply system in the intestine is discussed. The tissue-protective roles of HO-1 through the products of HO reaction in gastrointestinal pathophysiology are summarized, mainly based on our previous reports, focusing on protection of the barrier function. The beneficial effects of glutamine on the maintenance of intestinal integrity are also discussed.

## 2. Role of HOs in the Iron Supply System

**Figure 1:**
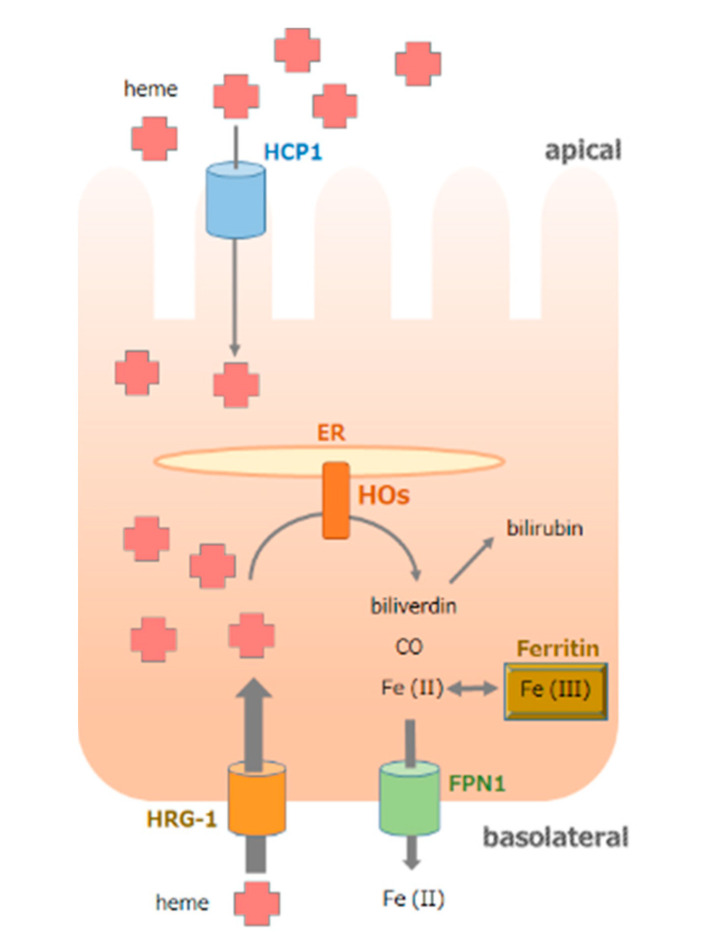
Heme utilization system as an iron-supplying system in gastrointestinal cells. In intestinal epithelial cells, heme is transported by heme carrier protein 1 (HCP1) from the apical membrane and by heme responsive gene-1 (HRG-1) from the basolateral membrane. The heme is more readily absorbed from the basolateral side than from the apical side. Since the catalytic sites of ER membrane-bound heme oxygenases (HOs) are in the cytosol, heme is degraded to biliverdin, which is rapidly reduced to bilirubin by biliverdin reductase in the cytosol. Fe (II) produced by the HO reaction is incorporated into ferritin or exported into tissue fluids via ferroportin 1 (FPN1).

The absorption of dietary iron, present mostly as heme and non-heme iron, occurs predominantly in the duodenum and upper jejunum, and is highly regulated [11]. The intestine can acquire heme from the basolateral side more effectively than from the apical side [12]. Different transporters localized in the apical and basolateral membranes have been suggested in polarized cultures of the human epithelial colorectal adenocarcinoma Caco-2 cell line, which is an established model of intestinal epithelial cells [13]. HO-1 induction, which is a marker of intracellular free heme content, is more pronounced when heme is applied at the basolateral side of cells [14]. Later, two heme transporters were reported, namely, heme carrier protein 1 (HCP1) [15] and heme responsive gene-1 (HRG-1) [16]. We previously reported that HCP1 is localized to the apical membrane and HRG-1 is localized to the basolateral membrane in polarized cells [17]. Localization studies supported a model in which cytosolic heme can be degraded by HOs, and the resulting iron is exported into tissue fluids via the iron exporter ferroportin 1, which is expressed in the basolateral membrane of enterocytes (Figure 1). In these processes, HOs play an important role because free heme itself acts as a pro-oxidant and generates potentially toxic moieties when its levels are abnormally increased [18]. Data from reported human cases of HO-1 deficiency and numerous studies using animal models suggest that HO-1 plays a critical role in various clinical settings involving excessive oxidative stress and inflammation [19].

## 3. Role of HO-1 in Protecting the Gastrointestinal Tract against Oxidative Injury

Inflammatory bowel disease (IBD) is a group of chronic inflammatory conditions affecting the colon and small intestine, and is one of the major health problems worldwide [20]. The disease is classified into Crohn’s disease (CD) and ulcerative colitis (UC), which are associated with a substantial patient burden and costs to healthcare systems [21]. Meanwhile, critically ill patients are susceptible to gut-origin sepsis [22]. HO-1 deficiency in humans has been implicated in susceptibility to inflammation [23], and HO-1 knockout mice showed higher mortality rates after exposure to endotoxins than wild-type mice [24]. These observations suggest that the upregulation of HO-1 is an adaptive response that protects the mucosa from oxidative injury in patients with gastritis and IBD [9].

The gut mucosa continuously experiences physiological stress. Intestinal tissues affected by IBD are known to overexpress HSP70 [25], the best-characterized member of the molecular chaperones that facilitates the refolding of stress-damaged proteins. HSPs are highly conserved proteins involved in the most basic cellular protection mechanisms and are induced by various stressors to provide cellular resistance against physical, chemical, and biological insults [26,27]. HSPs are tightly regulated; therefore, they are robustly induced in response to various stress conditions. Biopsies of patients with CD and UC showed increased mucosal HSP70 expression. It has been reported that the mucosal expression of HSP70 is enhanced in biopsies affected by CD and UC [25]. Exposure of the gastrointestinal mucosa to cytotoxic agents and cellular stresses induces HSPs, the physiological significance of which has been reviewed primarily from the viewpoint of molecular chaperones [28].

The functions of HSPs are not limited to their roles as molecular chaperones. HO-1 is the rate-limiting enzyme in heme catabolism [4,29] and is induced by various oxidative stresses through the activation of the transcription factor NF-E2-related factor 2 (NRF2) [30]. As NRF2 targets multiple genes involved in detoxification and antioxidation, it is considered a master regulator of the cellular responses to environmental stress [31]. We previously reported that HO-1 expression varies remarkably depending on the intestinal site in a rat model of sepsis. The upper intestine (duodenum and jejunum) is the principal site of HO-1 expression. In contrast, the enzyme is barely expressed and is not induced by the same treatments in the lower intestine (ileum and colon) [32]. In contrast to HO-1 expression, intestinal mucosal injury was more evident in the ileum and colon than in the duodenum and jejunum. The clear reciprocal relationship between HO-1 expression and intestinal tissue injury suggests the protective role of HO-1 against oxidative tissue injury. Indeed, the inhibition of HO activity by tin mesoporphyrin (SnMP) [33] augments both the expression of the tumor necrosis factor-α gene and mucosal epithelial cell injuries in the upper intestine. These findings suggest that more active HO-1 in the upper intestine than in the lower intestine plays a critical role in protecting intestinal epithelial cells from septic stress [32]. HO-1 likely plays an important role in IBD development, as it is upregulated in the affected colonic mucosa of patients with UC compared with their normal mucosa [34].

Our previous study demonstrated that orally administered nonsteroidal anti-inflammatory drugs (NSAIDs) induce apoptosis and produce lesions in the gastric mucosa. NSAIDs upregulate HO-1 in cultured gastric mucosal cells (primary cultures of guinea pig gastric mucosal cells) through the nuclear accumulation of NRF2. Under such conditions, NSAID-induced HO-1 upregulation protects the gastric mucosa by suppressing apoptosis. Upregulating HO-1 in the gastric mucosa at sufficiently high doses produces gastric lesions in rats [35]. This upregulation contributes to suppressing NSAID-induced apoptosis in gastric lesions [35]. Induction of HO-1 with cobalt protoporphyrin significantly ameliorated the pathogenesis of NSAID-induced gastric mucosal injury [36]. Therefore, non-toxic HO-1 inducers may be therapeutically beneficial as anti-ulcer drugs.

Previously, we reported, for the first time, that halothane-induced hepatotoxicity was attributed to reductive metabolite formation as well as an increase in the hepatic free heme concentration, which is a potent pro-oxidant. We demonstrated that the induction of HO-1 prior to exposure to halothane by heme treatment effectively prevented halothane-induced hepatotoxicity, indicating the importance of HO-1 induction in hepatic injury [37]. Similarly, hepatic HO-1 expression, mediated in part through a rapid increase in the microsomal free heme concentration (presumably derived from hepatic cytochrome P450), was markedly increased in a rat model of carbon tetrachloride-induced acute liver injury, which was exacerbated by the inhibition of HO activity by SnMP [38]. We also found that HO-1 induction in ischemic acute renal failure (IARF) played an important role in protecting renal cells against oxidative damage [39]. Tin chloride treatment before renal ischemia augmented the induction of HO-1 in renal epithelial cells of IARF rats. However, inhibition of HO activity by SnMP treatment abolished the beneficial effects of tin chloride pretreatment [40,41]. Recently, it was reported that intestinal ischemic postconditioning protects against kidney injury induced by intestinal ischemia reperfusion, which is likely mediated through NRF2 activation and the subsequent induction of HO-1 [42].

Hemorrhagic shock followed by resuscitation (HSR) causes oxidative stress that leads to tissue injury in various organs, including the lungs, liver, kidneys, and intestines. Excess amounts of free heme released from destabilized hemoproteins under oxidative conditions may constitute a major threat because it promotes the formation of reactive oxygen species (ROS). Previously, we investigated the relationship between the expression of HO-1 and tissue injury in the lungs, liver, and kidneys of HSR rats. It was found that tissues with higher HO-1 levels were better protected from oxidative tissue injury induced by HSR than those with lower HO-1 levels [43]. Our findings also suggested that HO-1 induced by heme pretreatment ameliorates HSR-induced lung injury, at least in part, through the DNA-binding activities of nuclear factor-κB and activator protein-1 [44]. We investigated the induction of HO-1 in intestines injured by HSR. HSR significantly increased the HO-1 levels in mucosal epithelial cells of the duodenum, jejunum, and colon, where mucosal inflammation and apoptotic cell death were far lower than those observed in the ileum. These findings indicate that HO-1 expression in the intestine is regulated in a site-specific manner following HSR. Moreover, HO-1 induction plays a fundamental role in protecting intestinal mucosal cells from oxidative damage induced by HSR [45].

HO-1 has been known to regulate the expression of many genes involved in the regulation of cell proliferation, migration, and invasion in many types of cancers [46,47,48,49,50]. Several lines of evidence have supported the implication of HO-1 in carcinogenesis and tumor progression [51]. HO-1 overexpression in cancer cells promotes proliferation and survival, partially through the induction of angiogenesis [52]. In gastrointestinal cancer, HO-1 expression in colorectal tumor cells was significantly higher than in normal cells, and an HO inhibitor increased chemotherapeutic sensitivity [46]. Recently, ferroptosis, a new form of programmed cell death regulated by iron accumulation [53], has been paid attention to, because several chemotherapeutic agents have been shown to induce ferroptosis in cancer cells [54,55]. The major intracellular iron source is the HO reaction; therefore, inducible HO-1 is an important enzyme, changing the intracellular iron distribution and leading to iron-dependent lipid peroxidation during ferroptotic cell death. HO-1 acts as a critical mediator in the induction of ferroptosis by operating cellular iron levels [56]. HO-1-mediated induction of ferroptosis suggested a novel chemotherapeutic strategy for cancer treatment [55,57].

In summary, HO-1 induction confers protection to tissues from further injury in various model systems, whereas abrogation of its induction accelerates cellular injury. Excess free heme, which is released from heme proteins under oxidative stress, such as oxidant stimuli, inflammation, exposure to xenobiotics, or ionizing irradiation, may constitute a major threat because it can promote ROS formation [58]. Therefore, HO-1 plays a pivotal role in protecting the tissues from oxidative injury [59].

## 4. Protective Effect of the Induction of HSPs on Gastrointestinal Barrier Function

An essential function of the intestinal epithelium is to maintain a selective barrier that excludes potentially harmful agents. The breakdown of the barrier is implicated in bacterial translocation, leading to sepsis and, consequently, in the pathogenesis of acute illnesses, such as multiple organ failure. The intestinal epithelial barrier is created by a single layer of epithelial cells lining the gastrointestinal tract [60]. The apical junctional complex comprises three junctions (from apical to basal) known as tight junctions (TJ), adherens junctions, and desmosomes [61]. TJ are selectively permeable barriers representing the rate-limiting step in paracellular transport.

The importance of the epithelial barrier in predisposition to IBD is supported by abnormal intestinal permeability in patients with CD [62]. In endotoxemia, intestinal barrier disruption is thought to contribute to the pathogenesis of heat stroke [63]. In contrast, a physiologically relevant increase in temperature causes an increase in intestinal epithelial TJ permeability via the induction of HSPs [64]. The master transcriptional regulators of HSPs are members of the heat shock transcription factor (HSF) family [65]. HSPs are induced by HSFs under various types of stress loading, which constitutes a pivotal part of cytoprotection against both exogenous and endogenous stressors [66]. HSF1 knockout mice presented with more advanced colitis than wild-type mice, whereas transgenic mice overexpressing HSP70 or HSF1 were characterized by an amelioration of the symptoms [67], demonstrating the protective actions of HSP70 and HSF1 against gastrointestinal damage. Similarly, pretreatment of rat intestinal epithelial cells with geranylgeranylacetone, a non-toxic HSP70 inducer with anti-ulcer action, ameliorated monocrolamine-induced oxidative injury [68]. Geranylgeranylacetone achieves its anti-ulcer effect by inducing HSPs, which are also protective against IBD-related colitis and lesions in the small intestine [69]. The mechanism by which HSP70 ameliorates intestinal disorders is based on suppressing pro-inflammatory cytokines under inflammatory conditions [67].

Alcohol abuse impairs the function of the intestinal barrier, which may enhance the translocation of bacterial toxins [70]. Previously, we reported the functional contribution of HSP70 to the maintenance of the intestinal epithelial barrier, focusing on barrier dysfunction caused by ethanol treatment, using filter-grown Caco-2 cells. Our results showed that early-phase induction of HSP70 significantly ameliorated barrier disruption [71]. In our study, ethanol exposure moderately activated HSF1 and increased its DNA-binding activity, resulting in a significant induction of HSP70 after injury. We demonstrated faster recovery when cells were subjected to heat stress to induce HSP70 expression prior to ethanol treatment. Therefore, we demonstrated a faster recovery from barrier dysfunction by loading cells with heat stress to induce HSP70 before ethanol treatment. Considering that the effect of ethanol on barrier function was caused by the production of the highly reactive metabolite acetaldehyde [72], our findings suggest that HSP70 may play an important role in the defense against barrier dysfunctions induced not only by endogenous ROS through thermal stress but also by exogenous chemical stress.

**Figure 2:**
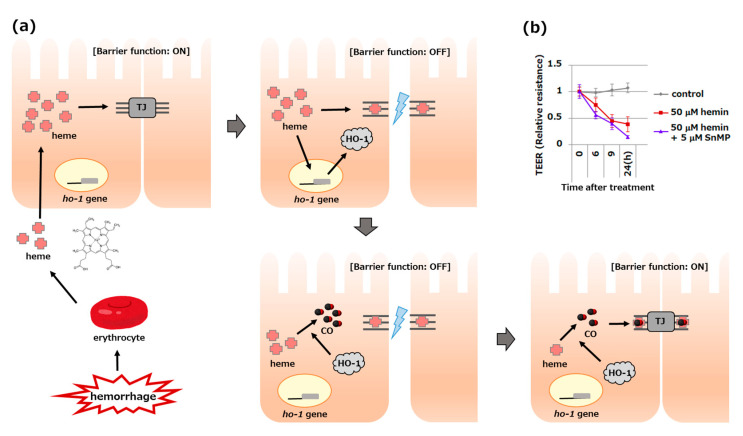
Possible role of HO-1 in protecting the barrier function of intestinal epithelial cells. (**a**) Free heme released from erythroids after intestinal hemorrhage causes an increase in intracellular free heme, which binds to components of the tight junction (TJ) complex, resulting in barrier dysfunction. Intracellular free heme also induces HO-1, reduces the intracellular free heme concentration, and produces carbon monoxide (CO) as a heme metabolite. As CO has a high affinity for heme proteins, it binds to the components of TJ, closing the barrier again by changing the protein’s configuration. (**b**) Measuring the trans-epithelial electrical resistance (TEER) in an experimental model using Caco-2 cells showed that hemin treatment disrupted the barrier function, which was aggravated by HO inhibition by SnMP.

Lower gastrointestinal hemorrhage is a common clinical problem in developed countries [73]. Colonic diverticular disease is associated with massive bleeding. We investigated changes in barrier function after intestinal hemorrhage using filter-grown Caco-2 cells [10]. We found that the epithelial barrier was disrupted by treatment with free heme, the local levels of which are expected to escalate in the tissue upon bleeding. Barrier function was impaired by heme treatment, primarily from the basolateral side, in a concentration-dependent manner. While heme treatment increased HO-1 expression in cells, inhibition of HO activity resulted in the aggravation of heme-induced barrier dysfunction, suggesting a protective function of HO-1 (Figure 2b). Supporting this possibility, the upregulation of HO-1 by pretreatment with a low heme concentration almost completely prevented heme-induced barrier dysfunction. The expression of occludin, which is a representative TJ protein, was significantly decreased in most injured cells, whereas HO-1 induction prior to heme-induced barrier disruption and pre-HO-1 induction maintained not only barrier function but also occludin levels [10]. We recently established HO-1 knockout Caco-2 cells and demonstrated that they were significantly vulnerable to heme-induced barrier dysfunction (unpublished data). Collectively, gastrointestinal hemorrhage causes oxidative stress via ROS production mediated by free heme, and barrier dysfunction caused by increased intracellular free heme, both of which are disrupted by HO-1 induction.

## 5. Role of CO in the Gastrointestinal Tract

Intestinal transplants are the least frequently performed because intestinal grafts typically suffer from ischemia/reperfusion (IR) injuries, resulting in prolonged intestinal dysfunction with a consequent loss of intestinal barrier function. Previous studies using an experimental rat model demonstrated that inhalation of CO gas, which was formed endogenously primarily through HO-mediated heme degradation, prevented IR injury in the transplanted intestine, an effect attributed to the anti-inflammatory and anti-apoptotic effects of CO [74,75,76]. These protective effects of CO have been confirmed in transplantation models of other organs, including the liver, heart, and kidney [77,78,79]. More specifically, studies conducted in the context of organ preservation have reported that intestinal grafts stored in University of Wisconsin solution in which CO gas was bubbled during cold storage before transplantation improved barrier function, downregulated the expression of several pro-inflammatory mediators, and markedly increased recipient survival [80].

Among the common physiologically relevant gases, nitric oxide, hydrogen sulfide, and CO are gaseous signaling molecules [81]. CO holds a unique position because of its remarkable chemical stability compared with the other two gaseous signaling molecules [82]. The cellular activity of CO has been linked to therapeutic benefits such as anti-inflammatory, anti-apoptotic, anti-hypertensive, anti-cancer, anti-diabetic, anti-malarial, and anti-bacterial effects, and it is protective against numerous indications, including IR injury, delayed graft function, organ injuries of various origins, and sickle cell disease [83,84,85]. Several experimental studies have shown that low levels of CO have potent cytoprotective effects during oxidative stress, hyperoxic lung injury [86], endotoxemia [87], surgical ileus [88,89], and arteriosclerosis [90]. The physiological significance of the CO/HO system has been demonstrated in numerous preclinical models, supporting the essential role of CO as a signaling molecule [91,92,93,94]. Previous studies have revealed that CO can modulate various physiological processes, including vasodilation, neurotransmission, platelet activation, and aggregation [95]. The specific functions, molecular targets, and participation of CO in signal transduction pathways have been clarified [83]. Owing to its stability and limited chemical reactivity with biological molecules distributed throughout the body and potential target sites, CO can exert its effects either locally or systemically by diffusing into remote tissues or organs. Therefore, CO can exert a wide range of protective actions under various pathological conditions [96,97,98,99].

CO plays a vital role in the physiology and pathophysiology of the gastrointestinal tract. CO derived from HO-1 protects the gastrointestinal tract from inflammation-induced damage [100]. Endogenously produced CO in the gastrointestinal tract establishes and maintains barrier function [101,102]. CO inhibits the production of pro-inflammatory cytokines in vitro and in vivo, and increases the expression of anti-inflammatory cytokines in the gastrointestinal tract [103].

The small intestine is a unique organ because it possesses vascular and luminal routes. Apart from the vessel walls, the layers of epithelial cells forming the mucosa and covering the inner part of the lumen are also highly susceptible to IR injury and thus are potential therapeutic targets. Therefore, exogenous CO is being explored as a therapeutic agent for various gastrointestinal disorders, including diabetic gastroparesis, postoperative ileus, organ transplantation, IBD, and sepsis [104]. However, CO gas administration for therapeutic purposes remains unclear because of the difficulties in carrying and controlling potential systemic toxicity. To address this problem, carbon monoxide-releasing molecules have been designed to carry and deliver controlled amounts of CO for therapeutic application [105].

CO potentially influences the biological activity of heme proteins. CO can inhibit the activities of cytochrome P450 and NADPH oxidase by forming complexes with them [106,107]. Progesterone receptor membrane component 1, a heme-containing protein that interacts with cytochrome P450, forms dimers through stacking interactions with heme molecules. CO interferes with the dimer by binding to heme [108]. Therefore, it has been hypothesized that barrier function disrupted by free heme in the Caco-2 bleeding model may be recovered by CO binding to the heme-binding proteins comprising the TJ. HO-1 induction enhanced the degradation of harmful free heme as well as CO production for the recovery of TJ function (Figure 2a). Regrettably, the molecular mechanism by which free heme disrupts barrier function has not yet been elucidated. Although significant progress has been made in uncovering the barrier dysfunction caused by abnormally increased intracellular free heme levels, most of these have been based on observational studies. Therefore, future research should focus on identifying the responsible protein(s) that interact with intracellular free heme, resulting in mechanisms that modulate the barrier function.

Moreover, there is strong evidence that CO is a messenger that regulates the gut microbiome and has beneficial effects on the host [109,110]. Enteric bacteria induce HO-1, which partly exerts homeostatic functions by increasing CO production and promoting the bactericidal activities of macrophages [111].

## 6. Role of Bilirubin in the Intestinal Tract

Bilirubin is one of the end products of the HO reaction. Previously, bilirubin was considered to be a potentially dangerous waste product of heme catabolism, because it was recognized as a sign of liver dysfunction or a toxic factor causing brain damage in newborns. However, the rediscovery of its antioxidant action [112,113] turned its position around, and it currently appears to have versatile functions, affecting biological activities with apparent clinical and even therapeutic consequences [114].

Bilirubin is a lipophilic molecule and diffuses into the bloodstream from the cells, binding to circulation albumin for hepatic delivery. After being absorbed into hepatocytes, bilirubin is conjugated with glucuronic acid by UDP glucuronosyltransferase 1A1 (UGT1A1) [115,116], facilitating excretion through the bile canaliculus, and is then directed into the gut, where bilirubin glucuronide is deconjugated by bacterial β-glucuronidases and then converted to urobilinoids [117], which is reabsorbed by intestine [118,119]. Unconjugated bilirubin is also reabsorbed and contributes to the circulating bilirubin pool [120]. Unconjugated bilirubin is known to be one of the most potent endogenous antioxidant substances, because it has strong ROS-scavenging, anti-oxidant, and cytoprotective properties [121,122,123]. Therefore, it is expected to contribute to protection against diseases associated with increased oxidative stress, such as cardiovascular diseases, diabetes, and colorectal cancer [124,125,126,127].

Recently, the effects of bilirubin on the gut microbiota have attracted considerable attention. The catabolism of bilirubin to urobilinoids may regulate a phylum or subspecies of bacteria in the gut that helps maintain homeostasis [128]. The observation that increasing plasma bilirubin decreases cholesterol levels suggests that the gut microbiota act as a contributor to lipid metabolism, affecting disease states [128]. Bilirubin was also shown to protect gut barrier integrity as well as to maintain microbiome richness and diversity [129]. It was reported that unconjugated bilirubin significantly decreased intestinal permeability and improved intestinal barrier function, ameliorating ulcerative colitis by inactivating the digestive proteases and inhibiting immune inflammation through the Toll-like receptor 4/nuclear factor-κB pathway [130]. It was also reported that the administration of biliverdin, which is rapidly reduced to bilirubin, to the small bowel attenuated transplantation-induced injuries, resulting in the recipient’s survival [131].

The beneficial associations of mildly elevated systemic bilirubin concentrations with diseases of civilization, including inflammatory diseases, have been reported [132,133,134]. The observed inverse correlation between serum bilirubin concentrations and a history of colorectal cancer supports further investigation of the potentially important chemopreventive function of bilirubin [124,135]. Gilbert’s syndrome is an inherited disease caused by decreased activity of UGT1A1. Unlike patients with Crigler–Najjar syndrome, in which UGT1A1 activity is barely detected, mildly elevated unconjugated bilirubin levels are observed in patients with Gilbert’s syndrome, who are usually asymptomatic. A low prevalence of ischemic heart disease was detected in these patients, leading to the presumption that chronic mild hyperbilirubinemia prevented the development of ischemic heart disease by increasing the serum antioxidant capacity [124,136]. A large cohort study was carried out, comparing all-cause mortality rates in those with and without Gilbert’s syndrome, and showing that the number of people with Gilbert’s syndrome in the general population is almost half that of people without Gilbert’s syndrome [137]. Although direct evidence has not been reported to show the beneficial effects of bilirubin on the intestinal tract in patients with Gilbert’s syndrome, it may be reasonable to suppose that the digestive system is also protected from oxidative stress though increased unconjugated bilirubin levels, because the direct transmural transport of unconjugated bilirubin from the blood to all parts of the intestinal lumen has been demonstrated [138].

## 7. Beneficial Effect of Glutamine on Gastrointestinal Disorders

Under conditions of critical illness, post-surgical stress, chronic inflammation, or starvation, glutamine is rapidly depleted in the body [139,140]. The intestinal tract is a key regulator of nitrogen handling following surgical stress in critically ill patients [22,140]. Glutamine is essential for intestinal metabolism, particularly following stress, and glutamine-enriched diets improve intestinal morphology and function [22,140,141,142].

Many investigators have reported that glutamine administration improves the therapeutic outcomes in experimental colitis models. Although the mechanism by which glutamine exerts its beneficial effects is not fully understood, it has been shown to improve intestinal barrier function [143] and anti-oxidative capacity [140,144] in colitis models. Glutamine depletion can increase gut permeability, promoting the translocation of luminal bacteria and toxins [140,142].

The non-nutritive effects of glutamine have been reviewed previously [140,145]. Evidence suggests that glutamine helps maintain intestinal mucosal integrity, particularly under stress conditions, such as radiation therapy [145,146] and experimental sepsis [145,147]. TJ protein expression and localization in Caco-2 cell monolayers rely on glutamine, which has been shown to maintain barrier function in intestinal cell culture monolayers [145,148]. Studies have shown that in Caco-2 model experiments, glutamine deprivation decreased the expression of claudin-1, occludin, and zonula occludens-1, three major proteins involved in intestinal epithelial TJ barrier function. Furthermore, it has been suggested that glutamine contributes to epithelial cell survival under stress conditions by inducing autophagy and by regulating the mechanistic target of rapamycin and p38 mitogen-activated protein kinase pathways [145,149].

**Figure 3:**
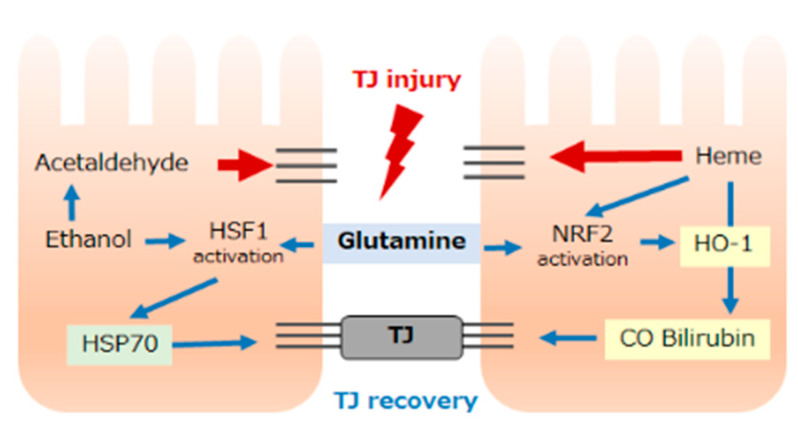
Putative mechanisms through which glutamine protects barrier function in the intestinal epithelium. Ethanol causes TJ injury via the metabolite acetaldehyde, followed by TJ recovery through heat shock protein 70 (HSP70) induced by heat shock factor 1 (HSF1) activation (left). In contrast, excess intracellular heme disrupts TJ, followed by HO-1 induction, which produces the metabolites CO and bilirubin, supporting TJ recovery. Supplementation with glutamine enhances HSF1 and NF-E2-related factor 2 (NRF2) activation, resulting in HSP70 or HO-1 induction, respectively.

Glutamine protects intestinal epithelial cells against physiological stress during stress-induced HSP expression [145,150,151]. Oral administration of glutamine in rats reportedly resulted in decreased gut permeability and improved survival following potentially lethal hyperthermia. In these animals, significantly enhanced expression of gut HSP70 was observed in the glutamine-supplemented group [145,152]. Glutamine can enhance lung Hsp70 expression and improve survival after cecal ligation and puncture-induced sepsis [145,153,154]. When the Caco-2 intestinal epithelial TJ barrier was disrupted by acetaldehyde, glutamine was reported to prevent an increase in permeability [64,145,155]. These observations strongly indicated a relationship between glutamine supplementation and HSP70 induction. We demonstrated that adding glutamine to the Caco-2 culture medium significantly improved barrier integrity after ethanol treatment. Glutamine supplementation increases the active form of HSF1 and subsequent HSP70 expression [71]. HSF1-null cells [71,154], as well as HSP70-null cells [59,141], showed no survival benefits following glutamine administration. Collectively, these observations indicate that glutamine protects the intestinal barrier function by modulating HSF1-mediated HSP70 expression (Figure 3).

We previously demonstrated that glutamine pretreatment significantly ameliorated intestinal tissue injury in endotoxemic rats, resulting in improved survival [71,156]. As the inhibition of HO activity completely abolished the beneficial effect of glutamine, the protective effect of glutamine was mediated by its lower intestine-specific induction of HO-1. We also demonstrated that glutamine significantly improved HSR-induced mucosal inflammation and apoptotic cell death in the rat ileum by inducing HO-1 [157].

The mechanism underlying the beneficial effect of glutamine in maintaining epithelial barrier function has been proposed to involve the maintenance of TJ protein expression and distribution [9,148,155]. We recently investigated the physiological role of HO-1 in intestinal barrier function, focusing on disorders associated with intestinal bleeding. In this model, glutamine was also beneficial for heme-induced barrier dysfunction [10,71]. We also demonstrated that nuclear NRF2 in heme-treated Caco-2 cells, in which marked HO-1 induction was observed, was significantly greater in cells grown in a glutamine-supplemented medium (unpublished data). Collectively, these observations indicate that the cytoprotective induction of HO-1 against an abnormally high level of intracellular free heme is mediated by the nuclear localization of NRF2 and that nuclear accumulation of NRF2 is enhanced by glutamine supplementation.
